# Molecular Docking and Screening Studies of New Natural Sortase A Inhibitors

**DOI:** 10.3390/ijms18102217

**Published:** 2017-10-23

**Authors:** Georgiana Nitulescu, Isabela Madalina Nicorescu, Octavian Tudorel Olaru, Anca Ungurianu, Dragos Paul Mihai, Anca Zanfirescu, George Mihai Nitulescu, Denisa Margina

**Affiliations:** Faculty of Pharmacy, “Carol Davila” University of Medicine and Pharmacy, Traian Vuia 6, 020956 Bucharest, Romania; georgiana.nitulescu88@gmail.com (G.N.); isabelanico77@gmail.com (I.M.N.); octavian.t.olaru@gmail.com (O.T.O.); ancaungurianu5@gmail.com (A.U.); dragosm1992@gmail.com (D.P.M.); anca.zanfirescu@umfcd.ro (A.Z.); denisa.margina@gmail.com (D.M.)

**Keywords:** sortase A, Gram-positive bacteria, MDR-bacteria, molecular docking, *Staphylococcus aureus*, anti-infective, *Daphnia magna*

## Abstract

To date, multi-drug resistant bacteria represent an increasing health threat, with a high impact on mortality, morbidity, and health costs on a global scale. The ability of bacteria to rapidly and permanently acquire new virulence factors and drug-resistance elements requires the development of new antimicrobial agents and selection of new proper targets, such as sortase A. This specific bacterial target plays an important role in the virulence of many Gram-positive pathogens, and its inhibition should produce a mild evolutionary pressure which will not favor the development of resistance. A primary screening using a fluorescence resonance energy transfer assay was used to experimentally evaluate the inhibitory activity of several compounds on sortase A. Using molecular docking and structure-activity relationship analyses, several lead inhibitors were identified, which were further tested for antimicrobial activity using the well diffusion test and minimum inhibitory concentration. The toxicity was assessed using the *Daphnia magna* test and used as a future screening filter. Three natural compounds were identified in this study as promising candidates for further development into therapeutically useful anti-infective agents that could be used to treat infections caused by multi-drug resistant bacterial pathogens which include sortase A in their enzymatic set.

## 1. Introduction

The multitude of infections caused by multi-drug resistant (MDR) bacteria has determined the need to discover or develop new ways to combat these microorganisms. Many bacterial species are able to adapt to the environment in which antibacterial agents are present using genetic mechanisms, mutations, or selection. Among the Gram-positive organisms, the most common are *Staphylococcus* sp. [1], *Enterococcus* sp. [2], and *Streptococcus* sp. [3], and from the Gram-negative class, we mention *Acinetobacter*, *Pseudomonas*, and various Enterobacteriaceae (including *Klebsiella*, *E. coli*, *Serratia*, and *Proteus*) [4].

Some strains of *Enterococcus* sp. and *Staphylococcus aureus* (*S. aureus*) have evolved to become highly aggressive nosocomial pathogens and are responsible for a wide range of infections such as endocarditis, urinary and biliary tract infections, bacteremia, meningitis, and wound infections [5,6]. The main issue surrounding these bacterial pathogens is that they often develop resistance to antibiotic drugs that target bacterial growth or viability. New strategies for developing the next-generation of antimicrobial therapeutics are being elaborated, one of which specifically targets virulence pathways that are non-essential for growth [7]. Unlike antibacterials which inhibit cell growth, anti-virulence therapies present a number of advantages since they produce a mild evolutionary pressure which will not favor the development of resistance, provide an increased repertoire of pharmacological targets, and generate agents with new mechanisms of action [8]. The possibility of numerous anti-virulence approaches is offered by the many virulence factors of *S. aureus* [9].

Among the factors regulating several central processes of bacterial biology (such as adhesion, colonization, and biofilm), are the transpeptidase enzymes known as sortases [10]. These enzymes catalyze a ligation reaction between a five amino-acid substrate motif (LPXTG) and oligoglycine nucleophiles [11] and play a significant role in the covalent attachment of surface proteins to the cell wall of bacteria [10].

The most studied sortase is sortase A (SrtA) from *S. aureus* due to the fact that this bacterium has increased pathogenicity and multi-drug resistance [12]. Its role in the virulence of many Gram-positive pathogens, including staphylococci, streptococci, enterococci, and *Listeria monocytogenes*, is proved by the fact that mutants lacking genes for SrtA display attenuated virulence, without affecting the growth of the bacteria [13]. The development and also the search for new inhibitors of SrtA use the *S. aureus* enzyme as a prototype.

An important source of bioactive molecules is represented by plants, and thus, they are being explored for the discovery and development of novel anti-virulence agents [14]. Of the many natural compounds that have been tested, flavonoids [15] and coumarins [16] are the most used for their antibacterial properties, but there are also anthraquinones [17] and alkaloids [18] with such properties. Due to their potential and accessibility, these compounds continue to be important sources of structural scaffolds for lead generation.

Even if the antibacterial activity is known for many of these compounds, their implications in anti-virulence is still uncertain. In this research, we continue our primary study of SrtA inhibitors [19] focusing on finding new inhibitors from natural sources. Molecular docking and structure-activity relationship analyses were performed to better understand the mechanism of inhibition and to reveal essential structural features. Antimicrobial activity was tested using the well diffusion test and the minimum inhibitory concentration (MIC) method, in order to find candidates with a minimum impact on bacterial cell growth. The toxicity was assessed using the *Daphnia magna* test as a prescreening method, thus considerably reducing the number of mammals required for future toxicity testing.

## 2. Results

### 2.1. Sortase A Activity Assay

A total of 56 natural compounds were chosen for a primary screening on SrtA activity based on their molecular diversity and on a set of rules derived from our previous research [19]. The candidates were small molecules with a low molecular flexibility and a minimum of two hydrogen bond acceptors. Chemically, 19 compounds belonged to the flavonoid family, six were coumarins, five were derivatives of cinnamic acid, five were alkaloids, three were anthraquinones, six belonged to the terpene family, and the rest had various scaffolds. Of the 56 natural compounds assayed, 22 compounds produced a significant inhibitory effect, of which seven compounds demonstrated good inhibitory effects on SrtA activity. The SrtA inhibitory effect was not correlated with the chemical family.

Based on the preliminary screening, a series of 11 compounds were selected to be tested at various concentrations in order to evaluate the inhibitory activity. The inhibitory activity was defined as the concentration of compound causing a 50% decrease in SrtA activity (IC50), relative to the negative control. The results are presented in Table 1. All the other compounds had no significant effect on SrtA at 10 μM.

**Figure 1:**
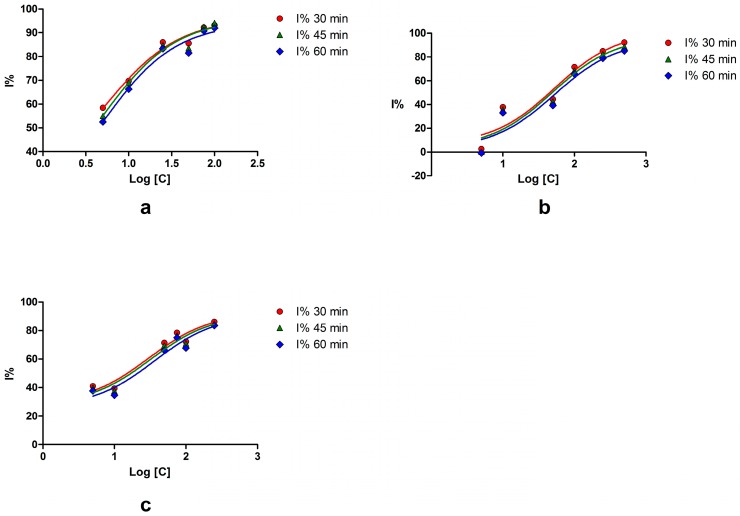
The inhibitory effect of natural compounds on SrtA after 30, 45, and 60 min. (**a**) myricetin; (**b**) palmatine chloride; (**c**) esculetin.

The IC50 value was only calculated for three compounds because the 50% inhibition threshold was not obtained for the others; the limited solubility in water hindered the use of the fluorescence resonance energy transfer (FRET) method. Myricetin showed the most potent activity on SrtA, its IC50 being eight-fold lower than esculetin, and eleven-fold lower than palmatine. For these three compounds, a good correlation between the concentrations and the inhibitory effect (Figure 1) on all moments of determination was observed (*r*^2^ > 0.9). Piperine, quercetin, and rhein demonstrated promising inhibitory effects at 10 μM, but their low solubility in water limited the testing at higher concentrations.

### 2.2. Protein-Ligand Interactions

**Figure 2:**
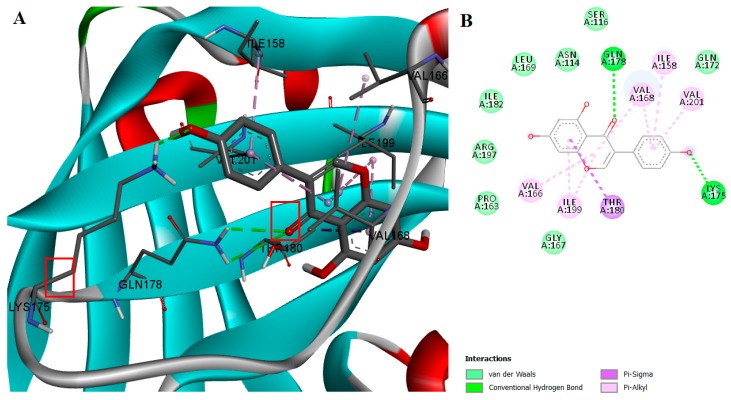
(**A**) Docked conformation and interactions of the genistein-SrtA complex; (**B**) 2D diagram of the predicted interactions; backbone of the protein is colored in grey, β-helices in turquoise, α-helices in red and loops in green. Ligand-protein interactions are colored depending on their type: conventional hydrogen bonds are colored in green, π-σ and π-alkyl interactions are colored in purple and light purple, respectively.

A computational docking algorithm was used to predict the relative binding affinities for a series of 16 inhibitors and to allow us to observe the structure-inhibitory action relationships. Among the tested compounds, genistein showed the lowest estimated free binding energy, and should thus have theoretically exhibited the highest transpeptidase inhibition activity. However, the SrtA inhibition assay demonstrated that genistein is inactive. The lack of correlation between in silico and in vitro results can be explained by analyzing genistein’s interaction with the enzyme. It was previously stated that the ionizable residues GLU105, SER116, THR180, THR183, ASP185, TRY187, LYS196, and ARG197 are situated proximal to CYS184 in the active site and thus facilitate transpeptidase enzymatic activity [20]. Genistein doesn’t participate in any hydrogen-bond interactions with the aforementioned residues, Even though genistein manifests a high binding affinity for SrtA and is involved in van der Waals and π-σ interactions with ARG197 and THR180, respectively (Figure 2), the interactions formed in the binding site do not seem to meet the requirements for inhibiting the transpeptidase activity.

**Figure 3:**
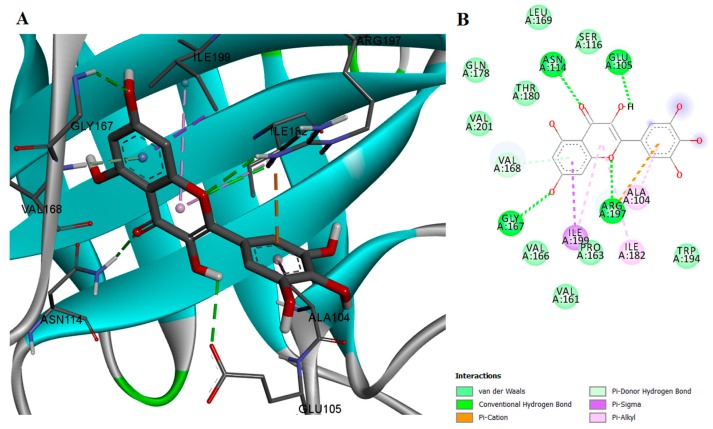
(**A**) Docked conformation and interactions of the myricetin-SrtA complex; (**B**) 2D diagram of the predicted interactions; backbone of the protein is colored in grey, β-helices in turquoise, α-helices in red and loops in green. Ligand-protein interactions are colored depending on their type: conventional hydrogen bonds are colored in green, π-σ and π-alkyl interactions are colored in purple and light purple, respectively.

On the other hand, myricetin exhibited both high inhibition activity and high binding affinity for SrtA, most likely due to its ability to form hydrogen bonds with two of the polar residues, GLU105 and ARG197. Moreover, myricetin also binds to GLY167, a residue which has been reported to play a major role in the SrtA inhibition activity of quercitrin [21]. Myricetin interacts through van der Waals forces with SER116, THR180, and other residues. The protein-ligand complex is stabilized by eight hydrophobic interacting residues and four types of π interactions (Figure 3). Furthermore, the docked conformation of myricetin is likely to hinder the interaction between the enzyme and its substrate, thus inhibiting SrtA activity in an orthosteric fashion. Myricetin and genistein have similar chemical structures, but there are two significant differences, the position of the phenyl substitution on the chromen-4-one scaffold and the fact that myricetin contains two additional hydroxyl groups, enabling its orientation in a more efficient manner in the SrtA binding site.

**Figure 4:**
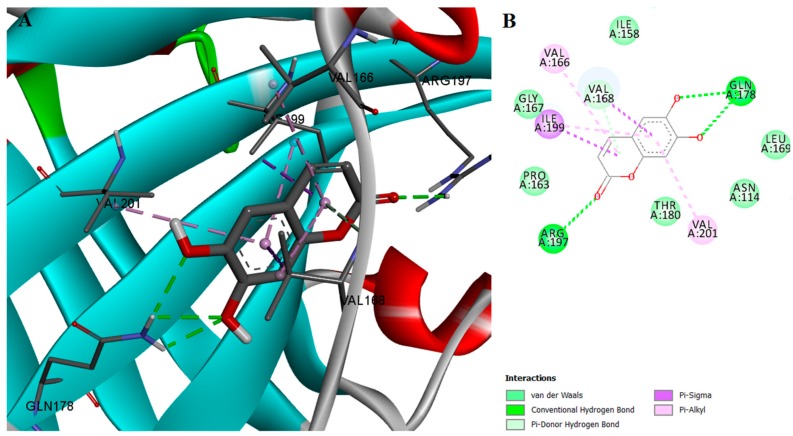
(**A**) Docked conformation and interactions of the esculetin-SrtA complex; (**B**) 2D diagram of the predicted interactions; backbone of the protein is colored in grey, β-helices in turquoise, α-helices in red and loops in green. Ligand-protein interactions are colored depending on their type: conventional hydrogen bonds are colored in green, π-σ and π-alkyl interactions are colored in purple and light purple, respectively.

Esculetin and palmatine chloride showed an inhibitory activity of SrtA six-fold and 10-fold lower than myricetin. Their binding affinities were also lower than the value predicted for myricetin. Like myricetin, esculetin can form conventional hydrogen bonds with ARG197 and a π-donor hydrogen bond with VAL168, which contribute to its ability to inhibit SrtA activity. Other interacting forces that can occur for both myricetin and esculetin are van der Waals interactions with THR180 and π-σ interactions with ILE199 (Figure 4).

**Figure 5:**
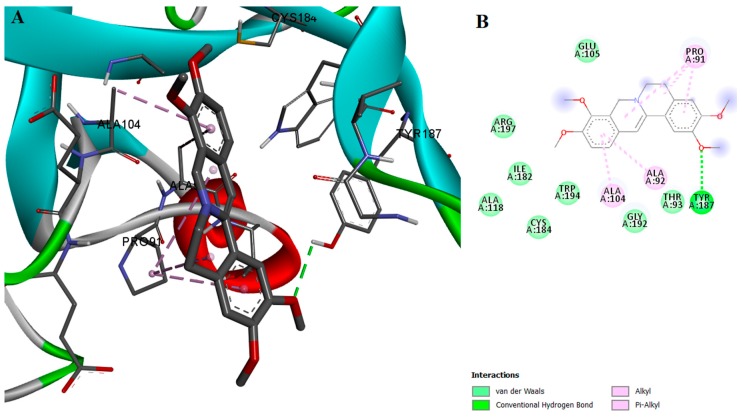
(**A**) Docked conformation and interactions of the palmatine-SrtA complex; (**B**) 2D diagram of the predicted interactions; backbone of the protein is colored in grey, β-helices in turquoise, α-helices in red and loops in green. Ligand-protein interactions are colored depending on their type: conventional hydrogen bonds are colored in green, π-σ and π-alkyl interactions are colored in purple and light purple, respectively.

Palmatine chloride has the highest binding energy amongst the active compounds, most likely due to its lack of hydrogen bond donor groups. Nonetheless, it can form a hydrogen bond with the ionizable residue TYR187 and participates in van der Waals interactions with GLU105, ARG197, and the catalytic residue, CYS184. The docked conformation is stabilized by alkyl and π-alkyl interactions and nine hydrophobic residues (Figure 5). Therefore, palmatine chloride can prevent the substrate from binding to the SrtA catalytic site.

**Figure 6:**
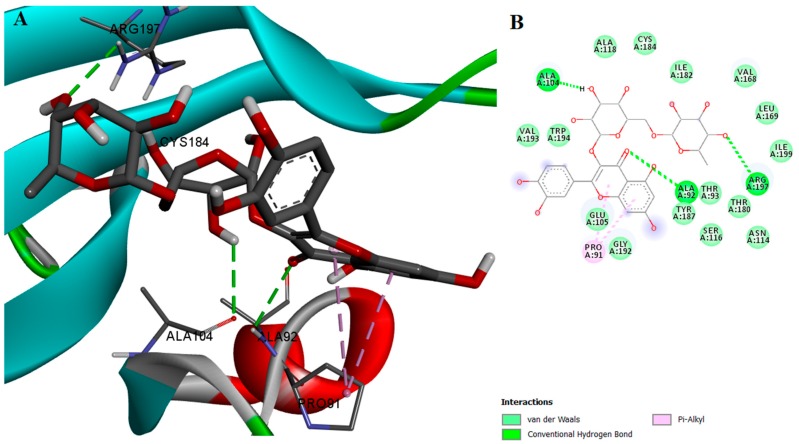
(**A**) Docked conformation and interactions of the rutin-SrtA complex; (**B**) 2D diagram of the predicted interactions; backbone of the protein is colored in grey, β-helices in turquoise, α-helices in red and loops in green. Ligand-protein interactions are colored depending on their type: conventional hydrogen bonds are colored in green, π-σ and π-alkyl interactions are colored in purple and light purple, respectively.

**Figure 7:**
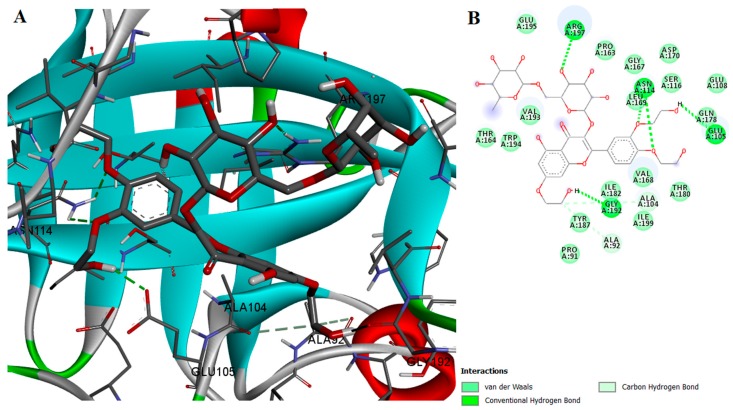
(**A**) Docked conformation and interactions of the troxerutin-SrtA complex; (**B**) 2D diagram of the predicted interactions; backbone of the protein is colored in grey, β-helices in turquoise, α-helices in red and loops in green. Ligand-protein interactions are colored depending on their type: conventional hydrogen bonds are colored in green, π-σ and π-alkyl interactions are colored in purple and light purple, respectively.

Although both rutin and troxerutin had binding affinities comparable to myricetin, their in vitro inhibition activity of SrtA was rather mediocre. Unlike myricetin, rutin and troxerutin are glycosylated and thus have a larger contact surface with the protein. Rutin can participate in hydrogen bonding with ARG197 and troxerutin can form the same bond type with ARG197 and GLU105. Both ligands interact with the enzyme through a large number of van der Waals and hydrophobic interactions and are likely to block the active site of the enzyme (Figure 6 and Figure 7). Possible reasons why these glycosylated flavonoids showed lower SrtA inhibition activities are that the protein-ligand complex might be prone to destabilization or the inability to enter the enzyme’s active site pocket due to their large molecular volume or high electrostatic repulsion. Nevertheless, rutin can be transformed in vivo by enzymatic deglycosylation into quercetin, which has been proven to inhibit SrtA enzymatic activity [22].

**Figure 8:**
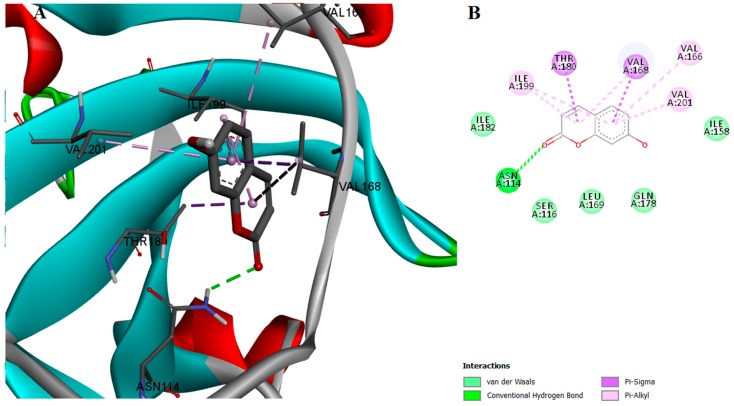
(**A**) Docked conformation and interactions of the umbelliferone-SrtA complex; (**B**) 2D diagram of the predicted interactions; backbone of the protein is colored in grey, β-helices in turquoise, α-helices in red and loops in green. Ligand-protein interactions are colored depending on their type: conventional hydrogen bonds are colored in green, π-σ and π-alkyl interactions are colored in purple and light purple, respectively.

Even though esculetin and umbelliferone have almost identical chemical structures and similar binding affinities, umbelliferone didn’t exhibit significant SrtA inhibition activity. Also, umbelliferone doesn’t participate in any hydrogen-bond interactions with the relevant ionizable residues, yet THR180 and SER116 are involved in π-σ and van der Waals interactions, respectively (Figure 8). Most likely, umbelliferone was unable to inhibit SrtA activity due to its lack of a second hydroxyl group on the aromatic ring.

### 2.3. Antibacterial Activity

Our molecular docking studies and the FRET-based assay evaluating the inhibitory effect on SrtA, indicated that the lead compounds should be further tested for antimicrobial activity. The antimicrobial activity of compounds was firstly screened by the well diffusion test and then subjected to the MIC method. The compounds were selected for the preliminary antibacterial screening if they produced a statistically significant SrtA inhibition, and are as following: artemisinin, chrysin, emodin, esculetin, genistein, myricetin, naringin, palmatine chloride, piperine, podophyllotoxin, quercetin, quinic acid, rhein, rutin trihydrate, troxerutin, and umbelliferone. Only emodin and rhein, two 1,8-dihydroxyanthraquinone derivatives, produced an observable antimicrobial effect on the tested bacteria strains. Table 2 shows the obtained values of the diameter inhibition zone for emodin and rhein.

Of the tested compounds, emodin and rhein exerted a potent inhibitory effect on Gram-positive *S. aureus* ATCC 25923, *S. epidermidis* ATCC 12228, and *B. cereus* ATCC 11778. None of the compounds exhibited antimicrobial activity against Gram-negative bacteria. *S. epidermidis* was found to be the most sensitive to the antimicrobial activity of rhein, while emodin had a significant effect on *S. aureus* ATCC 25923. Both compounds exerted moderate antimicrobial activity against *B. cereus* ATCC 11778.

Based on the preliminary assay, only emodin and rhein produced a significant effect to be tested for MIC determination. MIC values of emodin and rhein against the strains selected were obtained by well diffusion test screening (Table 3). It was noted that emodin had more inhibitory action against all bacterial strains than rhein. Even if the study on the bacterial growth influence was performed as a filter for a selective anti-virulence effect, the results of the antibacterial properties of the 1,8-dihydroxyanthraquinone derivatives could be developed.

### 2.4. Acute Toxicity Assessment Using Daphnia Magna

**Figure 9:**
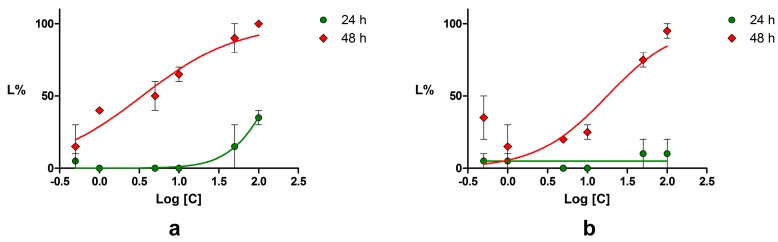

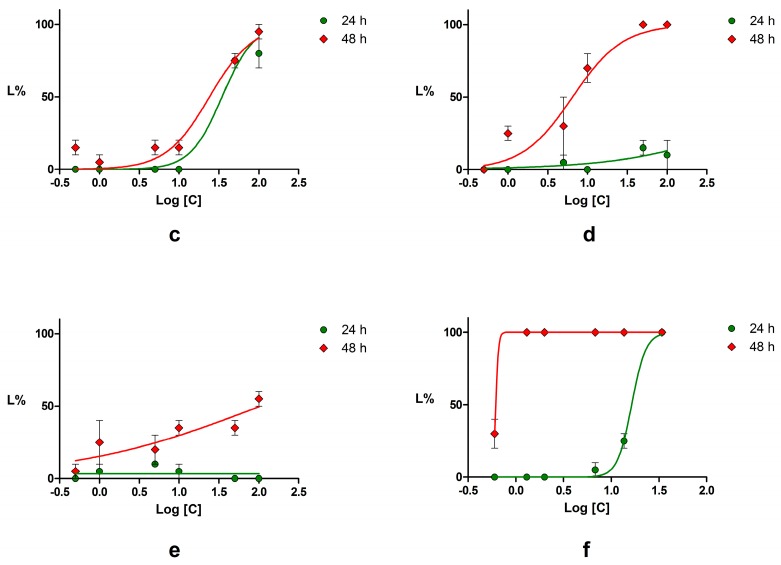
*Daphnia magna* lethality—logarithm of concentration curves at 24 and 48 h for: (**a**) piperine; (**b**) chrysin; (**c**) emodin; (**d**) podophyllotoxin; (**e**) umbelliferone; (**f**) potassium dichromate. Error bars represent standard error of the mean.

The majority of the compounds did not induce significant toxicity on *Daphnia magna*. Myricetin, palmatine chloride, naringin, rutin, esculetin, quercetin, and troxerutin induced a maximum of 30% lethality after 48 h. Rhein, arthemisin, and genistein induced a maximum toxicity between 40% and 60% at concentrations of 50 and 100 μM, respectively, but the results weren’t dose dependent and therefore median lethal concentration (LC50) couldn’t be calculated. A concentration-lethality dependence was obtained for piperine, chrysin, emodin, podophyllotoxin, and umbelliferone. For these compounds, the calculated LC50 values are presented in Table 4 and the lethality curves in Figure 9.

The highest toxicity on the crustacean model was produced by piperine, followed by podophyllotoxin, chrysin, emodin, and umbelliferone. The positive control, potassium dichromate, induced 100% toxicity after 48 h of exposure at concentrations in the 1.3–34 μM range. The calculated LC50 values after 24 h and 48 h demonstrated that all compounds produced a lower toxicity than the positive control.

## 3. Discussion

A chemically diverse set of 56 natural compounds were tested for their ability to inhibit SrtA activity, as potential anti-virulence agents. Myricetin, palmatine, and esculetin were identified as SrtA inhibitors, with promising IC50 values. Comparing these values with various known SrtA inhibitors, the compounds are in the first quartile [19]. Only a series of 2-phenylpyridazin-3-one derivatives are described in the literature, with IC50 values lower than myricetin [23].

The small number of hits hindered the obtaining of clear structure-activity relationships (SAR), but some structural patterns emerged as leads for future screening.

Myricetin, like quercetin and its 3-*O*-glycosides, hyperoside and rutin, belongs to the class of flavonols, all of which share a 3-hydroxyflavone backbone. Based on the in vitro study and on the protein-ligand interactions, the number and position of hydroxyl groups on the flavonol scaffold proved to be essential for SrtA inhibition. The IC50 value for quercetin was not calculated due to its very low solubility in water. The SAR study of the flavonoid group was therefore limited. The results of both the enzymatic assay and molecular docking indicates that the flavonoid aglycones are more potent towards SrtA than the corresponding glycosides. This observation is in concordance with our previous findings on SrtA inhibitor structural features, showing that the number of rotatable bonds should be less than or equal to 4 [19].

Esculetin is the lactone derivative formed by intramolecular cyclization of the caffeic acid, a derivative of cinnamic acid. It demonstrated good SrtA inhibition, while the caffeic acid and its methoxy-derivative, *trans*-ferulic acid, proved to be inactive. The results of the docking studies revealed the cause of this difference, the *cis*/*trans* conformation, and the importance of the hydrogen bonds between the carbonyl group and ARG197. The significance of the 6,7-dihydroxy-substitution on the chromen-2-one scaffold was demonstrated by the small inhibitory effect produced by both umbelliferone and coumarin. Esculin is esculetin 6-*O*-glucoside and also produced low SrtA inhibition because it lacks one essential hydrogen bonding donor, the 6-hydroxy group.

Palmatine chloride is a protoberberine alkaloid and has a low chemical similarity with all the compounds tested in this study. The results of the docking indicate an interaction with the catalytic center of the enzyme, but further research is needed to better understand this mechanism. 

Myricetin, palmatine chloride, esculetin, rutin, and troxerutin, presented the best inhibitory effect on SrtA and showed a low toxicity on *Daphnia magna*. The highest lethality induced by these compounds was 30% after 48 h of exposure at 100 μM, a value significantly higher than the IC50 values calculated for SrtA inhibition. The selected compounds also have the advantage of not influencing the Gram-positive bacteria growth, and therefore exhibit a low risk of inducing bacterial resistance.

Piperine and podophyllotoxin demonstrated promising SrtA inhibitions, but their low aqueous solubility and the high level of toxicity produced in the *Daphnia* test impedes their development as anti-virulence agents. Still, future molecular docking studies and SARs could reveal interesting new compounds.

## 4. Materials and Methods

### 4.1. Sortase A Activity Assay

The inhibitory activity of all of the compounds was determined by quantifying the increase in fluorescence intensity upon a cleavage 5-FAM/QXL^®^ FRET substrate using a SensoLyte^®^ 520 Sortase A Activity Assay Kit (Anaspec, San Jose, CA, USA). According to kit protocol, reactions were performed in a 96-well plate, with each well containing 10 μL test compound solution, 40 μL enzyme solution, and 50 μL sortase substrate solution. All compounds were dissolved in dimethyl sulfoxide (DMSO) and diluted with sterilized distilled water. The final concentration of DMSO was 1%. Each compound diluted in buffer to the tested concentration and was checked for intrinsic fluorescence. According to the kit protocol, we used the enzyme without the test compound as a control, a 1% DMSO control, and a substrate control. We used the kit inhibitor, 4-hydroxymercuribenzoic acid (HMB), as the positive control. Reactions were carried out for 1 h at room temperature and analyzed fluorometrically (SpectraMAX Gemini XS, CA, USA) at Ex/Em = 490 nm/520 nm. All reported values are the means of triplicate assays.

All reagents and solvents were purchased from commercial suppliers. The compounds used for screening were purchased from Sigma-Aldrich (St. Louis, MO, USA) and were as follows: Abietic acid (514-10-3), Aloin (1415-73-2), *trans*-Anethole (4180-23-8), Apigenin (520-36-5), Aristolochic acid I (313-67-7), Artemisinin (63968-64-9), Betulinic acid (472-15-1), Caffeic acid (331-39-5), Cantharidin (56-25-7), *trans*-Chalcone (614-47-1), Chelidonic acid (99-32-1), Chlorogenic acid (327-97-9), Chrysin (480-40-0), Coumarin (91-64-5), *m*-Coumaric acid (14755-02-3), *p*-Coumaric acid (501-98-4), Curcumin (458-37-7), Daidzein (486-66-8), Daidzein dimethylether (1157-39-7), Dihydrorobinetin (4832-33-6), Emodin (518-82-1), (−)-Epicatechin (490-46-0), Escin (6805-41-0), Esculetin (305-01-1), Esculin sesquihydrate (66778-17-4), Ethyl cinnamate (103-36-6), *trans*-Ferulic acid (537-98-4), Fisetin (345909-34-4), Genistein (446-72-0), Gibberellic acid (77-06-5), Glycyrrhizic acid ammonium salt (53956-04-0), Harmaline hydrochloride dehydrate (6027-98-1), Hesperetin (69097-99-0), Hesperidin (520-26-3), Hyperoside (482-36-0), *β*-Ionone (14901-07-6), Myricetin (529-44-2), Naringenin (67604-48-2), Naringin (10236-47-2), Oleanolic acid (508-02-1), Palmatine chloride (10605-02-4), Piperine (94-62-2), Podophyllotoxin (518-28-5), Quercetin (6151-25-3), Quinic acid (77-95-2), Resveratrol (501-36-0), Rhaponticin (155-58-8), Rhein (478-43-3), Rutin trihydrate (250249-75-3), Shikimic acid (138-59-0), Sinapic acid (530-59-6), Syringic acid (530-57-4), Troxerutin (7085-55-4), Umbelliferone (93-35-6), (+)-Usnic acid (7562-61-0), Vanillylacetone (122-48-5).

Nutrient broth (Oxoid Ltd., Basingstoke, UK) and Mueller-Hinton agar (Oxoid Ltd., Basingstoke, UK) were used as culture media. Gentamicin (Sigma-Aldrich, St. Louis, MO, USA) was used as a standard antibiotic. All standard bacteria were purchased from bioMérieux (Lyon, France). *Daphnia magna* Straus were maintained parthenogenetically at Carol Davila University (Department of Pharmaceutical Botany and Cell Biology; București, Romania).

### 4.2. Molecular Docking

#### 4.2.1. Protein and Ligand Preparation

The three-dimensional structure file of the *S. aureus* SrtA was downloaded from the RCSB PDB database and was prepared by adding missing polar hydrogen atoms using the Protoss tool provided by the Proteins Plus server [24]. All water molecules were removed and Gasteiger charges were assigned to the protein structure. 

The ligands’ chemical structures were retrieved from the PubChem database and were optimized with MarvinSketch 16.11.21 (ChemAxon Ltd., Budapest, Hungary). Furthermore, polar hydrogen atoms and Gasteiger charges were added to their structure. The 16 compounds were: artemisinin, chrysin, emodin, esculetin, genistein, myricetin, naringin, palmatine chloride, piperine, podophyllotoxin, quercetin, quinic acid, rhein, rutin trihydrate, troxerutin, and umbelliferone.

#### 4.2.2. Protein-Ligand Interaction Prediction

Molecular docking was performed using the AutoDock Tools 1.5.6 graphical user interface, which runs AutoDock Vina 1.1.2 software (Scripps Research Institute, San Diego, CA, USA). Autodock Vina is a docking platform which is faster and more accurate than the previous docking software, AutoDock 4 [25]. This newer version generates nine conformations (poses) of the protein-ligand complex which are displayed from lowest to highest binding free energy (ΔG).

Discovery Studio^®^ Visualizer 2016 (Accelrys Software Inc., San Diego, CA, USA) was used for the three-dimensional visualization and generation of two-dimensional plots of the protein-ligand interactions. Also, the number of residues which participated in hydrophobic interactions was predicted using the LigPlot Plus v.1.4 (European Bioinformatics Institute, Cambridge, UK) program.

### 4.3. Antibacterial Assay

Eight food-related microorganisms were used to evaluate the antimicrobial properties, including the Gram-positive *Staphylococcus aureus* ATCC 6538, *Staphylococcus aureus* ATCC 25923, *Staphylococcus epidermidis* ATCC 12228, *Enterococcus faecalis* ATCC 29212, *Bacillus cereus* ATCC 11778, and the Gram-negative *Escherichia coli* ATCC 8739, *Escherichia coli* ATCC 35218, and *Proteus mirabilis* ATCC 29245. The antibacterial assays were performed by the well diffusion [26] and broth microdilution method (ISO 20776-1) [27,28] in order to determine MIC.

Test microorganisms were stored in freezing conditions and activated by cultivation in Nutrient broth at 37 °C for 24 h. Overnight cultures were used to obtain the inoculum by transferring an amount of cells from the broth to a test tube containing 5 mL of demineralized water until a turbidity of 1–2 × 10^8^ colony-forming unit (CFU)/mL was achieved, equivalent to 0.5 McFarland standard [29]. Dilutions of the inoculum were cultured on nutrient agar supplemented with 5% sheep blood to verify the absence of contamination and the validity of the inoculum.

#### 4.3.1. Well Diffusion Test

Compounds were investigated by the agar well diffusion method. The plates containing Mueller Hinton agar medium were spread with 0.1 mL of the bacterial inoculum. The wells (6 mm diameter) were filled with 50 μL of the sample. The plates were incubated at 37 °C for 24 h. DMSO was used as a negative control and one standard antibiotic (gentamicin, 10 μg) as a positive control. Microbial inhibition was visually appreciated as the diameter of the inhibition zones surrounding the well and recorded in mm. All tests were run in triplicate for each combination of extract and microbial strain [26].

#### 4.3.2. Microdilution Test

Bacterial strains susceptible to compounds in the well diffusion assay were studied for their MIC values using 96-well microtiter plates [27,28]. The experiments were conducted using the reference method for testing the in vitro activity of the antimicrobial agents where MIC is the lowest concentration of an antimicrobial agent that, under defined in vitro conditions, prevents the appearance of visible growth of the microorganism after its incubation overnight. A total of 250 μL of the bacterial suspension in sterile demineralized water was added to 11 mL Mueller-Hinton broth (MBH) to a concentration of 5 × 10^7^ cells/mL. Compounds to be investigated were dissolved in MBH containing bacterial inoculum (5 × 10^4^ CFU/well) to achieve the two-fold serial concentrations ranging between 4-250 μL/mL [30].

The microplates were covered with an adhesive seal and incubated at 37 °C for 24 h. DMSO combined with MHB was used as the negative control for each bacterial strain. The plates were visually read under normal laboratory lighting using a manual mirror viewer. Two replicates were conducted for each compound and each bacterial strain.

### 4.4. Acute Toxicity Assessment Using Daphnia Magna

The *Daphnia magna* test was performed in 4 mL 12-tissue culture wells, using 10 daphnids/well, with each sample being tested in duplicate [31]. Lethality was recorded after 24 h and 48 h, considering the organisms that did not move their appendages for 30 s as dead. All experiments were conducted in the dark, in a plant growth chamber (Sanyo MLR-351 H, San Diego, CA, USA) at 25 ± 1 °C [32,33]. The bioassay was performed at concentrations ranging from 0.5 to 100 μM (0.5, 1, 5, 10, 50 and 100 μM) for each compound and, based on preliminary data, the positive control, potassium dichromate, at concentrations ranging from 0.2 to 10 μg/mL (0.2, 0.4, 0.6, 2.0, 4.0 and 10 μg/mL corresponding to 0.6, 1.3, 2.0, 6.8, 13.6 and 34.0 μM). The reference test with potassium dichromate was conducted to check the sensitivity of *Daphnia* to meet the validity criterion according to the OECD (The Organisation for Economic Co-operation and Development) guideline 202, if the LC50 of potassium dichromate at 24 h is ranging from 0.6 to 2.1 μg/mL [34].

### 4.5. Statistical Analysis

Statistical analysis of the inhibitory effect on SrtA and the *Daphnia magna* bioassay was performed using GraphPad Prism version 5.01 software (GraphPad Software, Inc., La Jolla, CA, USA). A student’s t-test (*p* < 0.05) was applied in order to analyze the results obtained from replicates. No statistical differences were found for three (inhibition on SrtA) or two (*Daphnia magna* bioassay) replicates. The inhibition (I %) and lethality percentage (L %) values were calculated and plotted against the logarithm of concentrations and the corresponding curves were calculated using the least squares fit method. The lethal concentration (LC50), which produces an L% value equal to 50, was determined by interpolation on the curves. Whenever the obtained results permitted, the upper and lower limits of the 95% confidence interval (95% CI) and the correlation coefficient (*r*^2^) were calculated.

## Figures and Tables

**Table 1:** The inhibitory effect on SrtA after 1 h of incubation.

Compound	Concentration (μM)	Inhibition (%)	IC50 (μM)	95% CI of IC50 (μM)
Myricetin	100	91.96	4.63	0.19–111.0
Esculetin	250	83.57	36.16	3.18–410.4
Palmatine chloride	500	85.29	52.84	3.46–805.7
Rutin trihydrate	500	28.71	NC *	NC *
Podophyllotoxin	50	26.40	NC *	NC *
Troxerutin	500	23.50	NC *	NC *
Quinic acid	500	19.56	NC *	NC *
Piperine	10	14.88	NT **	NC *
Emodin	100	10.14	NC *	NC *
Genistein	500	9.63	NC *	NC *
Quercetin	10	8.55	NT **	NC *
Rhein	10	7.01	NT **	NC *
Artemisinin	50	5.32	NC *	NC *
Umbelliferone	250	4.04	NC *	NC *
HMB	5	93.87	0.114	0.02–0.50

(*) NC—couldn’t be calculated due to the results obtained, (**) NT—not tested because of the low solubility, IC50—half maximal inhibitory concentration, 95% CI—95% confidence interval, HMB—4-hydroxymercuribenzoic acid.

**Table 2:** Antibacterial activity of the tested compounds ascertained by the well diffusion method.

Compound	*S. aureus* ATCC 6538	*S. aureus* ATCC 25923	*S. epidermidis* ATCC 12228	*E. faecalis* ATCC 29212	*B. cereus* ATCC 11778	*E. coli* ATCC 8739	*E. coli* ATCC 35218	*P. mirabilis* ATCC 29245
	Diameters of inhibition zone (mm), including diameter of well							
Gentamicin (10 μg)	17	21	29	12	24	21	20	20
Emodin (13.5 μg)	6	20	14	6	12	6	6	6
Rhein (14.2 μg)	6	12	30	6	15	6	6	6

**Table 3:** MIC values of the tested compounds (μg/mL).

Compound	*S. aureus* ATCC 25923	*S. epidermidis* ATCC 12228	*B. cereus* ATCC 11778
Emodin	8	8	16
Rhein	64	16	32
Gentamicin	2	2	4

**Table 4:** Results of the *Daphnia magna* bioassay.

Compound	LC50 (μM)		95% CI of LC50 (μM)	
	24 h	48 h	24 h	48 h
Piperine	144.60	3.45	82.26–254.1	1.89–6.27
Podophyllotoxin	NC *	6.47	NC *	4.01–10.46
Chrysin	NC *	17.68	NC *	7.73–40.44
Emodin	34.67	24.15	25.67–46.83	16.70–34.92
Umbelliferone	NC *	104.90	NC *	21.81–504.60
Potassium dichromate	16.39	0.62	4.15–5.59	NC *

(*) NC–couldn’t be calculated due to the results obtained, LC50–median lethal concentration.

## Abbreviations

CFU	colony-forming unit
CI	confidence interval
DMSO	dimethyl sulfoxide
FRET	fluorescence resonance energy transfer
HMB	4-hydroxymercuribenzoic acid
IC50	half maximal inhibitory concentration
LC50	median lethal dose
MBH	Mueller-Hinton broth
MDR	multi-drug resistant
MIC	minimum inhibitory concentration
SAR	structure-activity relationships
SrtA	sortase A
